# Annotations

**Published:** 1888-06-23

**Authors:** 


### Annotations.

**The Decay of Family Feeling.**

In a recent number of the *Fortnightly Review* Miss Frances Power Cobbe uttered an earnest plea for what she termed the "Education of the Emotions," and said that our modern materialistic tendencies made for the blunting and lowering of the emotional ideal. Even our philanthropy has for its aim only "barley-feeding and material ease;" and she instanced the growing habit among the poor of sending their sick to hospitals as a cause which tended to deaden the higher emotions, and consequent sense of responsibility, in those who by natural relationship should act as nurse in these cases. The illustration was not a happy one, for the wellbeing of the patient must be considered before the emotional development of the nurse; and it was exaggerated, for it is well known how often the poor refuse to part with their dear ones, even when removal to a hospital would obviously be for the benefit of the latter. Nevertheless there is a certain amount of truth in the accusation that our material charities are not always accompanied by a corresponding elevation of the moral tone, and that the very largeness of our philanthropic schemes tends to lessen the sense of individual responsibility. With all our philanthropy there is perhaps less of family charity existing in England now than ever before. The well-to-do and influential members of a family exert themselves to do,—what? To get their superannuated relatives, their old-maid aunts or sisters into some home or institution, where they will be relieved of all further responsibility or expense connected with them. We do not approve of—we protest most strongly against—the notion that a woman should live in idleness, sponging upon some male relation. Let every girl fit herself to earn her living, even though the necessity should never arise for her to live on her earnings. But we are now speaking of those to whom such advice would be given too late—to those who have been brought up to no calling, who, when they were thrown upon their own resources, struggled along while they had even a shadow of youth to help them, but were thrown "out of the running," as the incompetent always are with advancing age. It seems hard, no doubt, to the male kinsman that he should have to support, or help, these often unattractive women; but he should remember that he has had his college career, or his training in some remunerative business, to help him on in the world, while they have had a very inefficient education—stinted the more, perhaps, in order to give him greater advantages. In this aspect he probably owes them a debt.

**The "Daily Telegraph" and Our Bachelors.**

Imitation being the sincerest form of flattery, The Hospital feels inclined to make its best French bow to the *Daily Telegraph*, and say it is *trop flatteur*. Our humble annotation on the "Nursing of Bachelors" has been enlarged by it into a leader of more than a column in length; but though it quotes largely from our words, it does not bore its readers with any information about the name of the contemporary it condescendingly alludes to. Such things are beneath the dignity of "the largest circulation in the world." The *Globe* also swells the strain, under the jubilant title of "Good News for Bachelors," one week after the *Telegraph*. While agreeing with us in the main idea that nurses who would work at a smaller salary than most trained ones now ask would be a boon to the middle class in general—bachelors living in lodgings in particular—it carries our suggestion into regions The Hospital never dreamed of. It is well known that the present object of the *Daily Telegraph's* fondest anxiety is not bachelors, but spinsters, and our suggestion about the nursing of bachelors gives it a new idea on the question of "What to do with our girls." "Eureka!" cries the *Telegraph*, "let them announce themselves as ready to nurse unmarried men, and they will get a chance of occupation that may, as the advertisements say, become a permanency." The writer indeed says truly, that "we have the two sexes meeting and working together, but marriages do not always result." "But if they do," says the *Telegraph*, "why not?" Why not, indeed! If our bachelor patient wants, when recovered, to marry his nurse, and she is willing to take him, who would seek to forbid him? So much we allow gladly, but no more. The idea of a girl taking up nursing on the chance of meeting a husband in one of her patients is to us in the highest degree repulsive, and if practised habitually would degrade a profession which now holds an exceptionally high place in public esteem. Besides, how awkward it would be if our contemporary's dream was carried out to the full, and "The girls of London, who want a great deal of work, and some remuneration," were "enrolled as trained nurses, willing to attend unmarried men during illness"—perhaps widowers with a fair income and "no encumbrances" would not be objected to. Our idea was a society of nurses, for the most part not "girls," but thoughtful, motherly women, ready to go out nursing in any middle-class household, and attending to perhaps two or three cases at the same time, as the members of the East London and other nursing societies do,; and though we thought much of the bachelors, we did not mean them to have a monopoly of the benefit. But now what may we look forward to? The more self-respecting "bachelor's nurse" will have "No intentions" written on her card; while another will hint that though her terms are such and such, a reduction would be made in case of a proposal of marriage. But with the picture of these nurses there arises another question—Would the bachelors employ them?

**Lord Wolseley on Women's Thrift.**

Lord Wolseley presided at the annual general meeting of the depositors in the South-Eastern and Metropolitan Railways Savings Bank. In the course of his speech he made some highly practical remarks on *thrift*, which are well worth reproduction and emphasis. "He could not think," he said, "of any man who had made it a practice from early life of living within his income who was not successful. He was glad to see many women present, and that led him to remark that if there was thrift in a household it must be practised by the woman; but he had observed that there was a tendency among women of all classes to spend a great deal more on dress than their position justified. He was glad to hear some applause, but he was afraid it came from the men and not from the women. The difficulty with thrift was in the beginning; when the habit was once formed it lasted." The convictions expressed by Lord Wolseley are rather extensively entertained by men. The sterner sex, rightly or wrongly, consider that thrft is a weak point with their wives and daughters. As a set-off to these generally-received opinions may be mentioned the undoubted fact that the National Pension Fund for Nurses is being made use of in a degree altogether beyond the expectation of its most sanguine promoters. The very free and decidedly rash criticisms to which it has been subjected appear to have acted in quite the opposite direction from what was anticipated. It was stated at the annual meeting of The Hospitals Association that two hundred pension policies have already been issued, and that several hundred more have been applied for. These facts would seem to justify the contention that nurses, as a class, have reached a high level of intelligence and forethought, and that they may properly be considered as including among themselves some of the very best women of their own rank and degree. All the world knows that nurses as a body are self-denying and kind-hearted women. It will be a pleasing discovery to find that with their self-denial and gentleness they join forethought and thrift.

